# Melatonin Protects Human Renal Proximal Tubule Epithelial Cells Against High Glucose-Mediated Fibrosis via the Cellular Prion Protein-TGF-β-Smad Signaling Axis

**DOI:** 10.7150/ijms.42603

**Published:** 2020-05-18

**Authors:** Yong-Seok Han, Yeo Min Yoon, Gyeongyun Go, Jun Hee Lee, Sang Hun Lee

**Affiliations:** 1 Medical Science Research Institute, Soonchunhyang University Seoul Hospital, Seoul 04401, Republic of Korea.; 2 Institute of Tissue Regeneration Engineering (ITREN), Dankook University, Cheonan 31116, Republic of Korea.; 3 College of Science and Technology, Dankook University, Cheonan 31116, Republic of Korea.; 4 Department of Biochemistry, Soonchunhyang University College of Medicine, Cheonan 31151, Republic of Korea

**Keywords:** high glucose, fibrosis, melatonin, cellular prion protein, renal proximal tubule epithelial cells

## Abstract

Diabetes-mediated hyperglycemia is a major risk factor for renal fibrosis, resulting in the development of chronic kidney diseases. To address this issue, the effect of melatonin, which has an antioxidative potential, on renal fibrosis in human renal proximal tubule epithelial cells under high glucose conditions was investigated. Under high glucose conditions, the generation of reactive oxygen species was drastically increased in human renal proximal tubule epithelial cells, which lead to the inhibition of cell proliferation, enlargement of cell size, reduction of cell survival, and suppression of antioxidant enzyme activities. High glucose also increased the expression of transforming growth factor-β, leading to an increase in Smad2 phosphorylation. These fibrotic phenotype changes increased the expression of fibrosis-mediated extracellular matrix proteins, such as fibronectin, collagen I, and α-smooth muscle actin. In addition, the level of cellular prion protein (PrP^C^), which is associated with several biological processes, was decreased by exposure to high glucose conditions. Melatonin recovered the expression levels of PrP^C^ under high glucose conditions via phosphorylation of Akt, resulting in the prevention of high glucose-induced fibrosis. In particular, overexpression of PrP^C^ blocked the high glucose-mediated fibrotic phenotype change. These findings indicate that melatonin could be a powerful agent for treating hyperglycemia-induced renal fibrosis.

## Introduction

Diabetes is a progressive and chronic disease. The prevalence of chronic kidney diseases with diabetes is a significant public health burden in the United States population 1. Diabetic nephropathy is a complication with diabetes caused by glomerular filtration, glomerular hypertrophy, and renal fibrosis. In particular, hyperglycemia-induced renal fibrosis leads to end-stage renal disease, which is the major cause of death from diabetic complications. Despite several options for treating patients with diabetes, these options do not assure the reduction in the incidence of diabetic nephropathy 2. Therefore, it is important to find a novel strategy to protect diabetic complications in patients.

Transforming growth factor-β (TGF-β), which is a central mediator of fibrosis, promotes fibrosis through the induction of extracellular matrix (ECM) production and the activation of various intracellular signaling pathways 3. TGF-β is also a key regulator of the development of diabetic nephropathy 4. TGF-β activates the downstream mediators, Smad2 and Smad3, which then induce the expression of several pro-fibrotic genes, such as collagen I, collagen IV, integrin, and fibronectin 5. Excessive deposition of ECM proteins causes renal malfunction and failure. Although these results indicate that regulation of TGF-β protein is an important strategy for protecting renal fibrosis, TGF-β is also involved in various biological processes, such as apoptosis, autophagy, cell proliferation, differentiation, and immune response 6. Thus, many studies have tried to investigate a strategy for regulating a balanced level of TGF-β to protect against the adverse effects of targeting TGF-β 6.

Melatonin is an endogenous hormone secreted from the pineal gland, as well as from other tissues, including bone marrow, liver, gut, ovary, and testes 7, 8. Accumulating evidence has shown that melatonin regulates physiological responses, such as sleep, circadian rhythms, immune defense, and neuroendocrine actions 7, 9. Melatonin (N-acetyl-5-methoxytryptamine) is also a pleiotropic and multifunctioning indoleamine, which plays roles in antioxidative, antitumor, immunoregulatory activities 10. Thus, melatonin has been reported to modulate a wide spectrum of molecular pathways, including apoptosis, autophagy, oxidative stress, inflammation, cellular injury, and fibrosis 11-14. In the context of anti-fibrosis, melatonin inhibits apoptotic and necrotic changes, and infiltration of inflammatory cells; suppresses the activation of fibrogenic effector cells; attenuates the expression of fibrosis-inducing factors, in particular, TGF-β; and decreases the deposition of ECM proteins 14. Therefore, to investigate whether melatonin protects renal cells against hyperglycemia-induced fibrosis, the effect of melatonin on high glucose-induced fibrosis in renal proximal tubule epithelial cells was assessed. In addition, the expression of cellular prion protein (PrP^C^), which is associated with several biological processes such as stress protection, cellular differentiation, mitochondrial homeostasis, and cell signaling pathways 15, was investigated in renal proximal tubule epithelial cells under high glucose conditions.

## Materials and Methods

### Cell culture

Human renal proximal tubule epithelial cell line (TH1) was purchased from Kerafast (Boston, MA, USA). Cells were grown in Dulbecco's modified Eagle's medium (HyClone, Logan, UT, USA) supplemented with 10% (v/v) fetal bovine serum (HyClone), 100 U/mL penicillin (HyClone), and 100 mg/mL streptomycin (HyClone). Cells were incubated in a humidified incubator at 37 °C in an atmosphere of 95% air and 5% CO_2_.

### Cell proliferation assay

Cell proliferation was examined using a bromodeoxyuridine (BrdU) incorporation assay. TH1 cells were cultured in 96-well culture plates (5,000 cells/well). TH1 cells were exposed to D-glucose (0, 10, 25, and 50 mM) for a fixed period of 48 h, or to 50 mM D-glucose for 48 h with or without melatonin (1 μM) pretreatment with luzindole (1 μM). BrdU incorporation was performed using an enzyme-linked immunosorbent assay (ELISA) colorimetric kit (Roche, Basel, Swiss). To perform the ELISA, 100 μg/mL BrdU was added to TH1 cell cultures and incubated at 37 °C for 3 h. An anti-BrdU antibody (100 μL) was added to TH1 cell cultures and incubated at 25 °C for 90 min. Then, 100 μL of a substrate solution was added, and 1 M H_2_SO_4_ was employed to stop the reaction. Light absorbance was measured for each sample using a microplate reader (BMG Labtech, Ortenberg, Germany) at 450 nm.

### Cell survival assay

TH1 cells were cultured in 96-well culture plates (5,000 cells/well) with D-glucose (0, 10, 25, and 50 mM) for 48 h. Cell survival was determined using a modification of the 3-(4,5-dimethylthiazol-2-yl)-2,5-diphenyltetrazolium bromide (MTT) assay, which is based on the conversion of the tetrazolium salt 3-(4,5-dimethylthiazol-2-yl)-5-(3-carboxymethoxy-phenyl)-2-(4-sulfophenyl)-2-tetrazoluim to formazan by mitochondrial dehydrogenase. Absorbance of formazan was measured using a microplate reader (BMG Labtech) at 450 nm.

### LDH release assay

Lactate dehydrogenase (LDH) release was determined using an LDH cytotoxicity detection kit (Takara Bio, Tokyo, Japan). Briefly, TH1 cells were cultured in 96-well culture plates (5,000 cells/well), and treated with 50 mM D-glucose for 48 h, with or without melatonin (1 μM) pretreatment with luzindole (1 μM) to assess the LDH activity. Next, the reaction mixture (100 μL) was added to each well and incubated at 25 °C for 30 min. The absorbance was determined using a microplate reader (BMG Labtech) at 490 nm.

### DHE staining

To measure superoxide anion levels in cultured TH1 cells, the cells were incubated with 10 μM Dihydroethidium (DHE) (Sigma-Aldrich, St. Louis, MO, USA) for 30 min at 37 °C. After washing with phosphate buffered saline (PBS) three times, samples were visualized by flow cytometry (Sysmex, Kobe, Japan).

### SOD activity

TH1 cells were harvested, and protein was isolated using a radioimmunoprecipitation assay (RIPA) lysis buffer (Thermo Fisher Scientific, Waltham, MA, USA). The cell lysates (protein 500 µg) were treated with superoxide dismutase (SOD), and the signal was immediately measured each minute for 15 min using an ELISA reader (BMG Labtech) at 450 nm.

### Catalase activity

TH1 cells were seeded in 100 mm tissue culture plates and grown to 70% to 75% confluence. After washing twice in PBS, cells were resuspended in lysis buffer (1% Triton X-100 in 50 mM Tris-HCl [pH 7.4], containing 150 mM NaCl, 5 mM EDTA, 2 mM Na_3_VO_4_, 2.5 mM Na_4_PO_7_, 100 mM NaF, and protease inhibitor cocktail (Thermo Fisher Scientific)). Samples were incubated for 30 min on ice and were centrifuged at 14,000 rpm for 30 min at 4 °C. After measuring the protein concentration of the supernatant fraction, catalase activity was measured using a Catalase Assay Kit (Sigma-Aldrich) according to the manufacturer's instructions.

### TGF-β ELISA

TGF-β protein levels were evaluated in TH1 cells that were treated with 50 mM D-glucose for 48 h, with or without melatonin (1 μM) pretreatment with luzindole (1 μM) using a TGF-β ELISA kit (Komabiotech, Seoul, Korea). Total proteins (500 μg) from each group of TH-1 cell lysates were subjected to these experiments. All ELISAs were performed in triplicate. Expression levels of TGF-β were quantified by measuring absorbance at 450 nm on a microplate reader (BMG Labtech).

### Western blot analysis

The total cellular protein of TH1 cells was extracted using RIPA lysis buffer (Thermo Fisher Scientific). Cell lysates were subjected to sodium dodecyl sulfate-polyacrylamide gel electrophoresis, and the proteins were transferred to polyvinylidene fluoride membranes (Sigma-Aldrich). Membranes were blocked with 5% skim milk and incubated with primary antibodies against manganese-dependent superoxide dismutase (MnSOD; Santa Cruz Biotechnology, Dallas, TX, USA), p-Smad2 (Novus Biological, Centennial, CO, USA), Smad7 (R&D system, Minneapolis, MN, USA), fibronectin (Thermo Fisher Scientific), p-Akt (Santa Cruz Biotechnology), PrP^C^ (Santa Cruz Biotechnology), Collagen I (Santa Cruz Biotechnology), E-cadherin (Santa Cruz Biotechnology), α-smooth muscle actin (α-SMA; Santa Cruz Biotechnology), and β-actin (Santa Cruz Biotechnology). After incubation of membranes with peroxidase-conjugated secondary antibodies (Santa Cruz Biotechnology), bands were visualized using enhanced chemiluminescence reagents (Thermo Fisher Scientific) in a dark room. For quantification of band intensity, each band intensity was analyzed by ImageJ software (http://rsb.info.nih.gov/ij/). The expression levels of proteins were determined relative to the expression of β-actin.

### Detection of PrP^C^ in cell lysates

Concentrations of PrP^C^ in TH1 cells incubated with 50 mM D-glucose for 48 h, with or without melatonin (1 μM) pretreatment with luzindole (1 μM), were determined using a commercially available ELISA kit (Lifespan Biosciences, Seattle, WA, USA). Total proteins (500 μg) from each group of TH1 cell lysates were subjected to these experiments. All ELISAs were performed in triplicate. Expression levels of PrP^C^ were quantified by measuring absorbance at 450 nm on a microplate reader (BMG Labtech).

### Statistical analysis

All results are expressed as the mean ± the standard error of the mean (SEM). One-way analysis of variance followed by Tukey's post hoc test was used for multiple comparisons. Differences were considered to be statistically significant if *p* < 0.05.

## Results

### Melatonin increased the proliferation of renal proximal tubule epithelial cells under high glucose conditions

**Figure 1:**
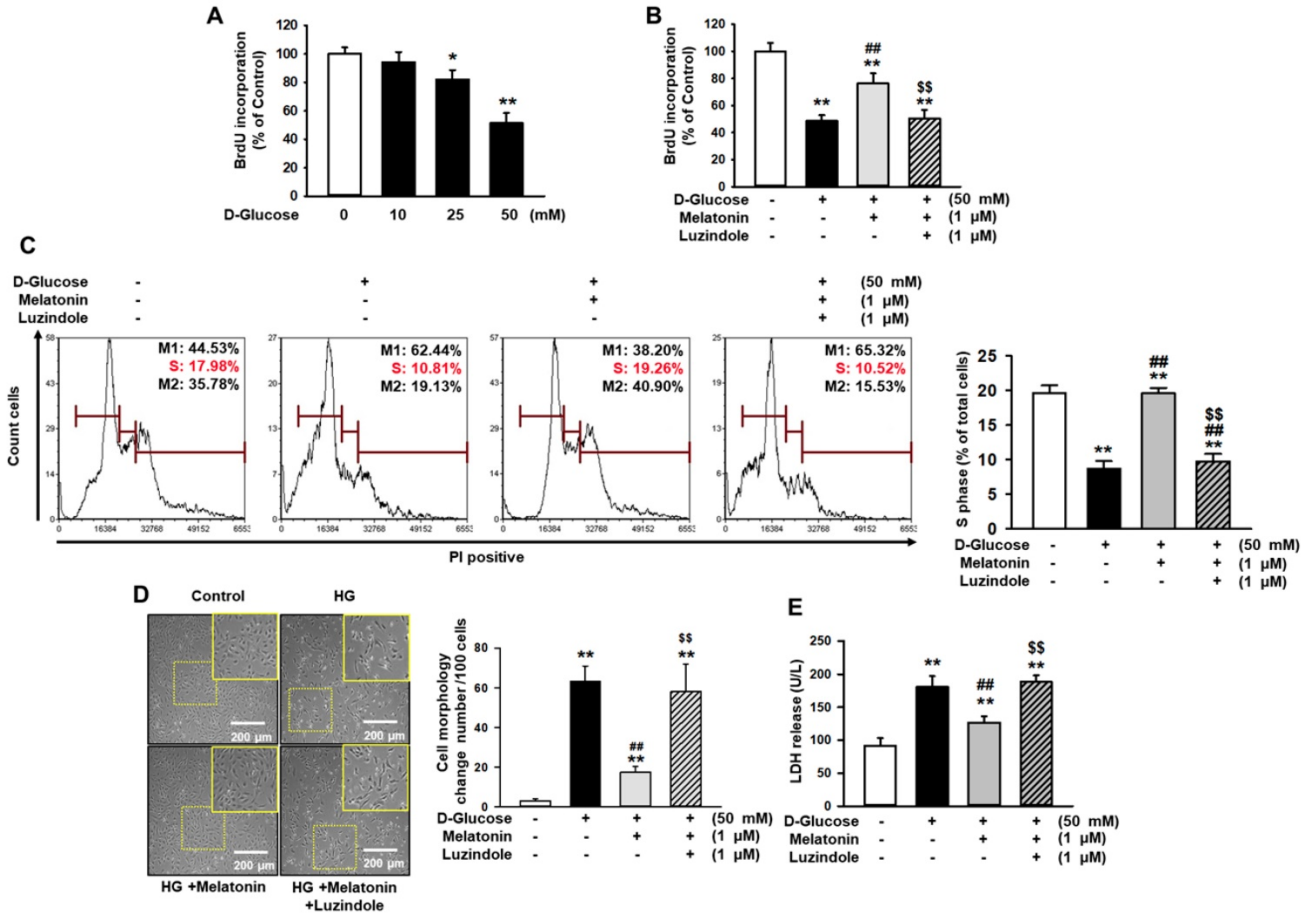
** Melatonin inhibits the proliferation, enlargement, and reduction in the survival rate of renal proximal tubule epithelial cells under high glucose conditions.** (A) BrdU incorporation in human renal proximal tubule epithelial cell lines (TH1) after treatment with glucose at different concentrations (0, 10, 25, and 50 mM). Values represent the mean ± SEM (n = 3). ^*^p < 0.05, ^**^p < 0.01 vs. control. (B) BrdU incorporation in human renal proximal tubule epithelial cells after treatment with melatonin under high glucose conditions (50 mM). Luzindole acts as a selective melatonin receptor antagonist. Values represent the mean ± SEM (n = 3). ^**^p < 0.01 vs. control, ^##^*p* < 0.01 vs. TH1 cells + D-glucose, ^$$^*p* < 0.01 vs. TH1 cells + D-glucose + Melatonin. (C) Cell cycle analysis in human renal proximal tubule epithelial cells after treatment with melatonin under high glucose conditions (50 mM). Cell proliferation is quantified as the percentage of S phase in each group. Values represent the mean ± SEM (n = 3). ^**^p < 0.01 vs. control, ^##^*p* < 0.01 vs. TH1 cells + D-glucose, ^$$^*p* < 0.01 vs. TH1 cells + D-glucose + Melatonin. (D) The morphology of human renal proximal tubule epithelial cells after treatment with melatonin under high glucose conditions (HG; 50 mM). The bar graph represents the number of cells that showed changes in morphology (cell enlargement). Values represent the mean ± SEM (n = 5). ^**^p < 0.01 vs. control, ^##^*p* < 0.01 vs. TH1 cells + D-glucose, ^$$^*p* < 0.01 vs. TH1 cells + D-glucose + Melatonin. (E) LDH release analysis in human renal proximal tubule epithelial cells after treatment with melatonin under high glucose conditions (HG; 50 mM). Values represent the mean ± SEM (n = 3). ^**^p < 0.01 vs. control, ^##^*p* < 0.01 vs. TH1 cells + D-glucose, ^$$^*p* < 0.01 vs. TH1 cells + D-glucose + Melatonin.

To investigate the effect of melatonin in renal proximal tubule epithelial cells under high glucose conditions (50 mM), cell proliferation was assessed by BrdU incorporation analysis. The proliferation and survival of renal proximal tubule epithelial cells were decreased in a glucose dose-dependent manner (Figure 1A and Supplementary Figure 1A). Under high glucose conditions (50 mM), cell proliferation and survival were significantly increased with melatonin treatment, compared to control (Figure 1B, 1C, and Supplementary Figure 1B). In addition, luzindole, which is a selective melatonin receptor antagonist, blocked this effect (Figure 1B, 1C, and Supplementary Figure 1B). In addition, cell was treated with mannitol (50 mM) as an osmotic control, resulting that mannitol (50 mM) did not decrease the cell survival (Supplementary Figure 2). Morphological analysis showed that high glucose significantly increased cell size (Figure 1D). The LDH release assay, which assesses cytotoxicity, also showed that high glucose significantly induced cytotoxicity (Figure 1E). Melatonin also protected high glucose-mediated cell morphological alteration and high glucose-induced cytotoxicity in a melatonin receptor-dependent manner (Figure 1D and 1E). These results indicate that melatonin attenuated high glucose-induced damages in renal proximal tubule epithelial cells.

### Melatonin protected renal proximal tubule epithelial cells against high glucose-induced fibrosis

**Figure 2:**
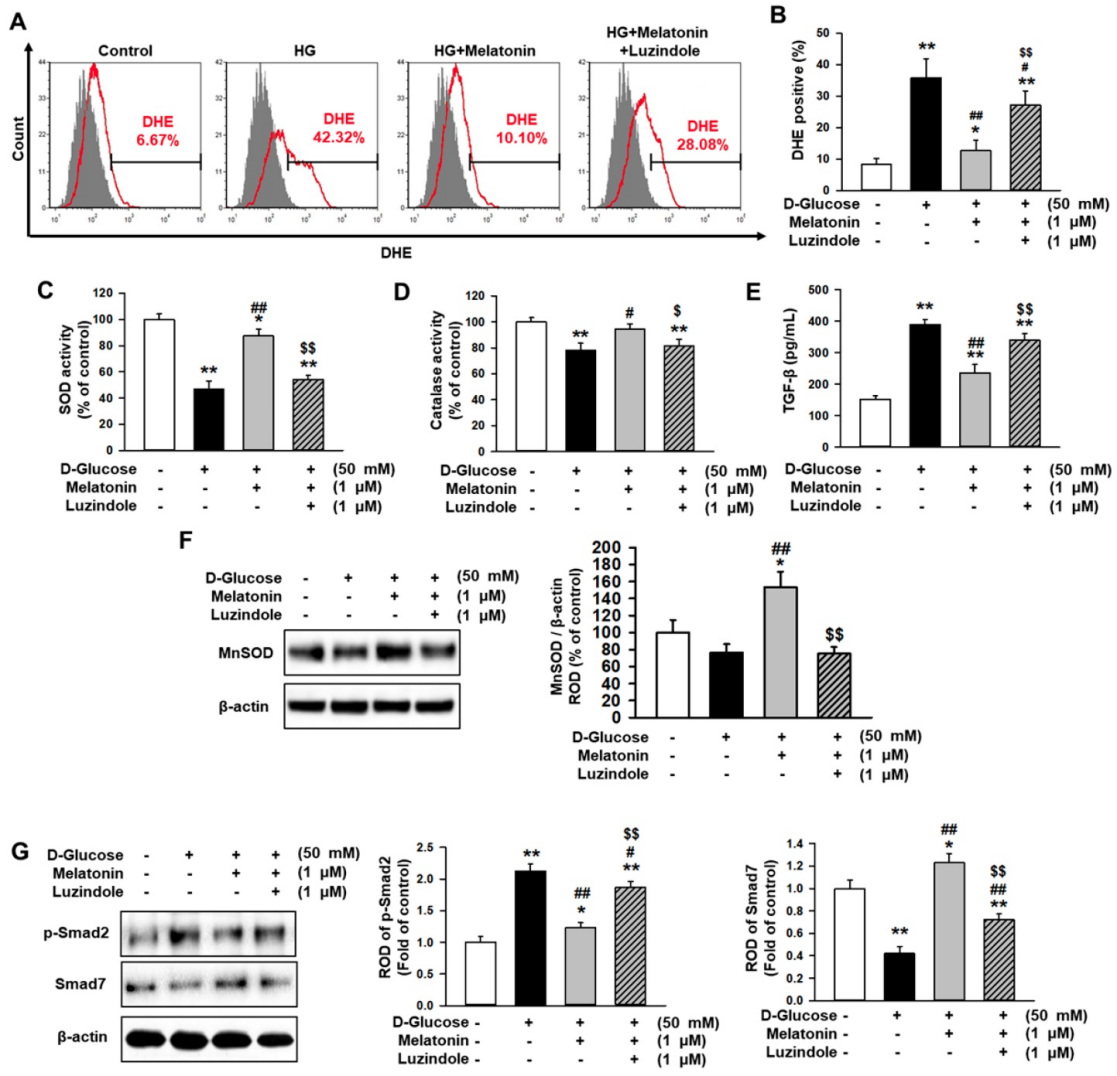
** Melatonin decreases the alteration of high glucose-induced fibrotic phenotypes.** (A) Flow cytometry analysis for DHE staining in human renal proximal tubule epithelial cells after treatment with melatonin under high glucose conditions (HG; 50 mM). (B) Quantification of flow cytometry analysis for DHE staining. Values represent the mean ± SEM (n = 5). ^**^p < 0.01 vs. control, ^#^*p* < 0.05, ^##^*p* < 0.01 vs. TH1 cells + D-glucose, ^$$^*p* < 0.01 vs. TH1 cells + D-glucose + Melatonin. (C-E) SOD activity (C), catalase activity (D), and TGF-β expression (E) in human renal proximal tubule epithelial cells after treatment with melatonin under high glucose conditions (50 mM). Values represent the mean ± SEM (n = 3). ^*^p < 0.05, ^**^p < 0.01 vs. control, ^#^*p* < 0.05, ^##^*p* < 0.01 vs. TH1 cells + D-glucose, ^$^*p* < 0.05, ^$$^*p* < 0.01 vs. TH1 cells + D-glucose + Melatonin. (F) Expression of MnSOD in human renal proximal tubule epithelial cells after treatment with melatonin under high glucose conditions (50 mM). The level of expression is determined relative to the expression of β-actin. Values represent the mean ± SEM (n = 3). ^*^p < 0.05 vs. control, ^##^*p* < 0.01 vs. TH1 cells + D-glucose, ^$$^*p* < 0.01 vs. TH1 cells + D-glucose + Melatonin. (G) Expression of p-Smad2 and Smad7 in human renal proximal tubule epithelial cells after treatment with melatonin under high glucose conditions (50 mM). The levels of expression are determined relative to the expression of β-actin. Values represent the mean ± SEM (n = 3). ^*^p < 0.05, ^**^p < 0.01 vs. control, ^#^*p* < 0.05, ^##^*p* < 0.01 vs. TH1 cells + D-glucose, ^$$^*p* < 0.01 vs. TH1 cells + D-glucose + Melatonin.

To investigate whether melatonin inhibits the generation of reactive oxygen species (ROS) in renal proximal tubule epithelial cells under high glucose conditions, flow cytometry analysis using DHE staining was performed. The production of ROS was markedly increased in treatments with high glucose compared to the control, whereas treatment with melatonin suppressed the production of ROS under high glucose conditions (Figure 2A and 2B). In addition, luzindole blocked the inhibitory effect of melatonin on ROS production (Figure 2A and 2B). Furthermore, high glucose significantly decreased antioxidant components, such as SOD and catalase activities, but melatonin prevented the decrease associated with high glucose exposure (Figure 2C and 2D). The expression of TGF-β was also elevated under high glucose conditions, whereas melatonin blocked the high glucose-induced expression of TGF-β (Figure 2E). These results indicated that melatonin inhibited the alteration of fibrosis-associated phenotypes under high glucose conditions. Moreover, western blot analysis for MnSOD showed that the augmentation of SOD activity by treatment with melatonin was associated with the increase in MnSOD expression (Figure 2F). To further explore whether melatonin is involved in the regulation of fibrosis-associated signaling pathways in renal proximal tubule epithelial cells under high glucose conditions, the expression of p-Smad2, which is associated with pro-fibrotic signaling, and Smad7, which is involved in anti-fibrotic signaling, was assessed. High glucose conditions significantly increased the phosphorylation of Smad2 and drastically decreased the expression of Smad7 (Figure 2G). However, melatonin mitigated the activation of p-Smad2 and recovered the levels of Smad7 under high glucose conditions (Figure 2G). These effects were inhibited by treatment with luzindole (Figure 2G). These findings indicate that melatonin inhibits high glucose-induced fibrosis in renal proximal tubule epithelial cells through the regulation of the antioxidant effects and the fibrosis-associated signaling pathways.

### Melatonin decreased the expression level of fibronectin under high glucose conditions

**Figure 3:**
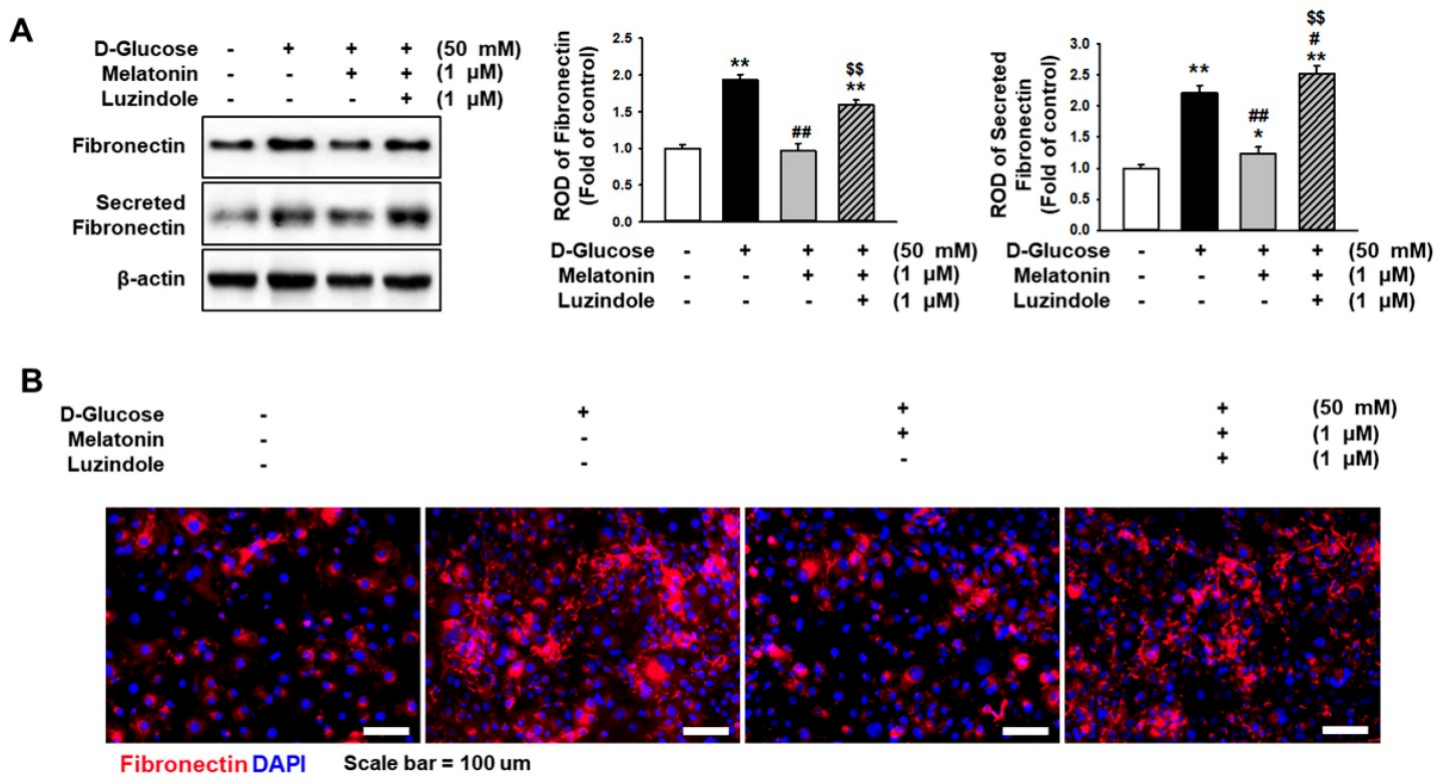
** Melatonin inhibits the high glucose-induced expression of fibronectin.** (A) Expression of fibronectin in human renal proximal tubule epithelial cells after treatment with melatonin under high glucose conditions (50 mM). The levels of expression are determined relative to the expression of β-actin. Values represent the mean ± SEM (n = 3). ^**^p < 0.01 vs. control, ^#^*p* < 0.05, ^##^*p* < 0.01 vs. TH1 cells + D-glucose, ^$$^*p* < 0.01 vs. TH1 cells + D-glucose + Melatonin. (B) Immunofluorescence staining for fibronectin (red) in human renal proximal tubule epithelial cells after treatment with melatonin under high glucose conditions (50 mM) (n = 3). Nuclei were stained with DAPI (blue). Scale bar = 50 μm.

Excessive expression of fibronectin induces fibrosis in several tissues. To determine whether melatonin regulates the expression of fibronectin in renal proximal tubule epithelial cells under high glucose conditions, the expression of fibronectin in renal proximal tubule epithelial cells and the secretion of fibronectin were analyzed. Under high glucose conditions, the expression of fibronectin and the secretion of fibronectin in renal proximal tubule epithelial cells were markedly increased (Figure 3A), whereas treatment with melatonin inhibited the intracellular and extracellular expression levels of fibronectin (Figure 3B). In addition, immunofluorescence staining for fibronectin showed that melatonin decreased the high glucose-induced expression of fibronectin (Figure 3B). These effects were blocked by the use of luzindole (Figure 3A-3C). These results indicate that melatonin suppresses the intracellular and extracellular expression of fibronectin in renal proximal tubule epithelial cells under high glucose conditions.

### Melatonin increased the expression of PrP^C^ under high glucose conditions through activation of Akt

**Figure 4:**
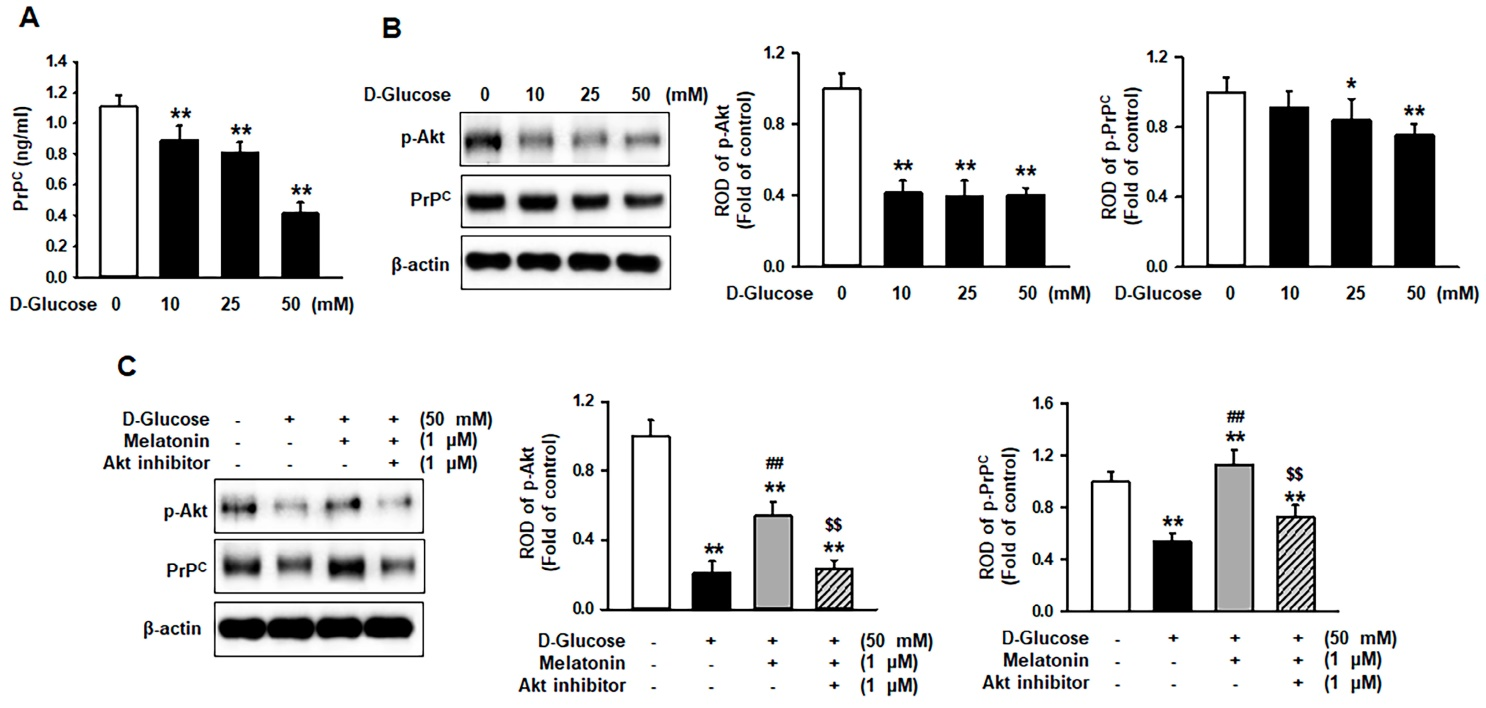
** Melatonin increases the expression of PrP^C^ under high glucose conditions via activation of the Akt signaling pathway.** (A) ELISAs for PrP^C^ in human renal proximal tubule epithelial cells after treatment with glucose at different concentrations (0, 10, 25, and 50 mM). Values represent the mean ± SEM (n = 3). ^**^p < 0.01 vs. control. (B) Expression of p-Akt and PrP^C^ in human renal proximal tubule epithelial cells after treatment with glucose at different concentrations (0, 10, 25, and 50 mM). The levels of expression are determined relative to the expression of β-actin. Values represent the mean ± SEM (n = 3). ^*^p < 0.05, ^**^p < 0.01 vs. control. (C) Expression of p-Akt and PrP^C^ in human renal proximal tubule epithelial cells after treatment with melatonin under high glucose conditions (50 mM). The levels of expression are determined relative to the expression of β-actin. Values represent the mean ± SEM (n = 3). ^**^p < 0.01 vs. control, ^##^*p* < 0.01 vs. TH1 cells + D-glucose, ^$$^*p* < 0.01 vs. TH1 cells + D-glucose + Melatonin.

Cellular prion protein (PrP^C^) is known to regulate several biological processes 15. To demonstrate whether high glucose affects the expression of PrP^C^ in renal proximal tubule epithelial cells, the expression of PrP^C^ in renal proximal tubule epithelial cells under high glucose conditions was induced. The level of PrP^C^ was significantly decreased in a glucose dose-dependent manner (Figure 4A). Previous studies have shown that high glucose suppresses the phosphorylation of protein kinase B (Akt) in an ROS-sensitive manner 16 and that the expression of PrP^C^ is regulated through the activation of Akt 17. Thus, the activation of Akt and the expression of PrP^C^ in renal proximal tubule epithelial cells after treatment with glucose at various concentrations (0, 10, 25, and 50 mM) was assessed. The phosphorylation was significantly decreased with the treatment of glucose (10, 25, and 50 mM), and the expression of PrP^C^ was drastically inhibited in a glucose dose-dependent manner (Figure 4B). To further explore whether melatonin increases the Akt phosphorylation and PrP^C^ level in renal proximal tubule epithelial cells under high glucose conditions, the phosphorylation of Akt and the expression of PrP^C^ was assessed under high glucose (50 mM) conditions. Treatment with melatonin recovered the high glucose-induced inhibition of PrP^C^ via activation of Akt (Figure 4C). These findings have shown the mechanism by which melatonin increases the expression of PrP^C^ through the upregulation of Akt phosphorylation under high glucose conditions.

### Melatonin prevented high glucose-induced fibrosis through the upregulation of PrP^C^

**Figure 5:**
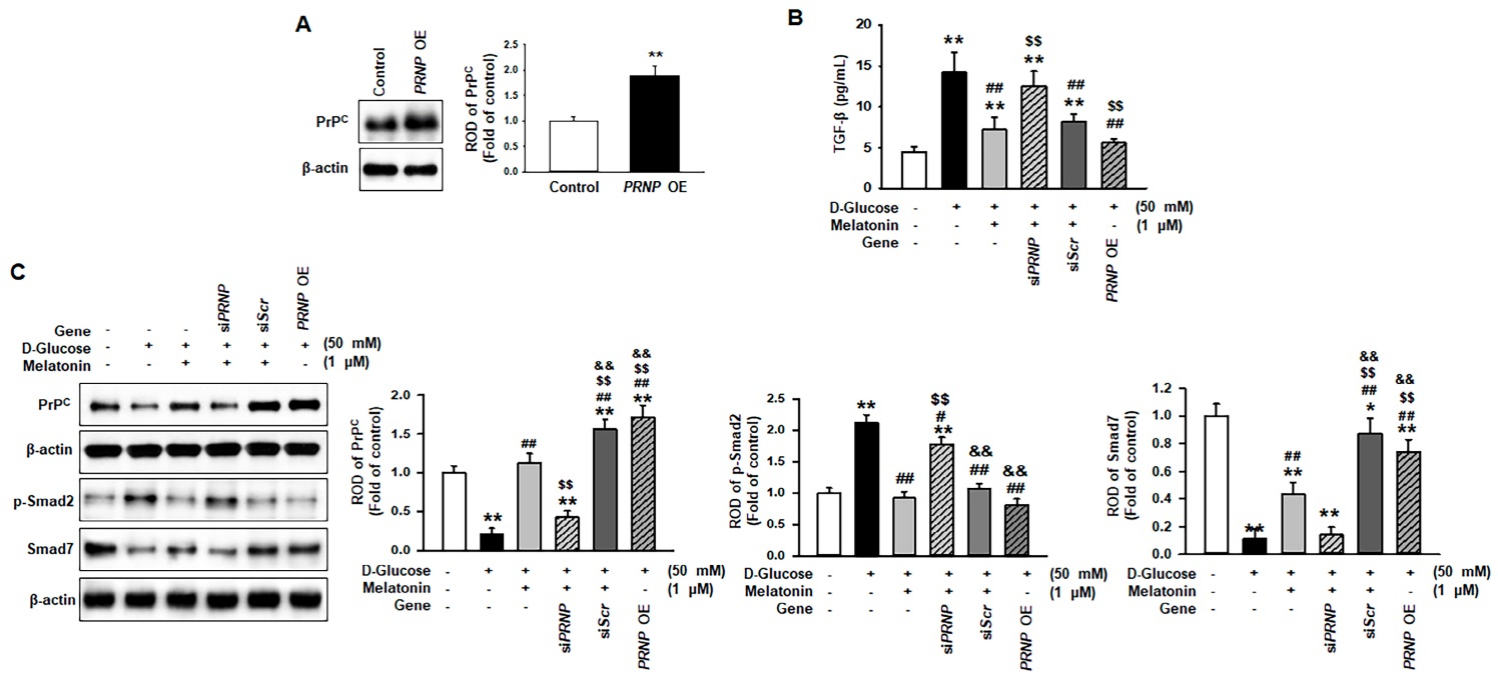
** Melatonin suppresses the activation of the fibrosis-mediated signaling pathway through the upregulation of PrP^C^.** (A) Expression of PrP^C^ in renal proximal tubule epithelial cells overexpressing the *PRNP* gene (*PRNP* OE), which encodes PrP^C^. The level of PrP^C^ expression is determined relative to the expression of β-actin. Values represent the mean ± SEM (n = 3). ^**^p < 0.01 vs. control. (B) ELISA analysis for TGF-β in renal proximal tubule epithelial cells under high glucose conditions (50 mM) after treatment with melatonin. Values represent the mean ± SEM (n = 3). ^**^p < 0.01 vs. control, ^##^*p* < 0.01 vs. TH1 cells + D-glucose, ^$$^*p* < 0.01 vs. TH1 cells + D-glucose + Melatonin + scrambled siRNA (siScr). (C) Expression of PrP^C^, p-Smad2, and Smad7 in renal proximal tubule epithelial cells under high glucose conditions (50 mM) after treatment with melatonin. The levels of expression are determined relative to the expression of β-actin. Values represent the mean ± SEM (n = 3). ^**^p < 0.01 vs. control, ^#^*p* < 0.05, ^##^*p* < 0.01 vs. TH1 cells + D-glucose, ^$$^*p* < 0.01 vs. TH1 cells + D-glucose + Melatonin, ^&&^*p* < 0.05 vs. TH1 cells + D-glucose + Melatonin + *PRNP* siRNA (si*PRNP*).

To reveal whether melatonin inhibits high glucose-induced fibrosis in renal proximal tubule epithelial cells in a PrP^C^ expression-dependent manner, PrP^C^ expression was upregulated or downregulated, and the change in high glucose-induced fibrotic phenotypes was confirmed (Figure 5A). Under high glucose conditions, the level of TGF-β was significantly decreased via PrP^C^ expression after treatment with melatonin (Figure 5B). In addition, the overexpression of *PRNP* inhibited the high glucose-induced elevated expression of TGF-β in renal proximal tubule epithelial cells that were not treated with melatonin (Figure 5B). Furthermore, under high glucose conditions, melatonin decreased the phosphorylation of Smad2 and increased the expression of Smad7 through the upregulation of PrP^C^ (Figure 5C). The overexpression of *PRNP* also mitigated the activation of the high glucose-induced fibrosis-mediated signaling pathway in cells that were not treated with melatonin (Figure 5C). These findings suggest that melatonin-induced PrP^C^ could be a key molecule for preventing high glucose-mediated fibrosis.

### Melatonin suppressed the expression of fibrotic markers under high glucose conditions through the upregulation of PrP^C^

**Figure 6:**
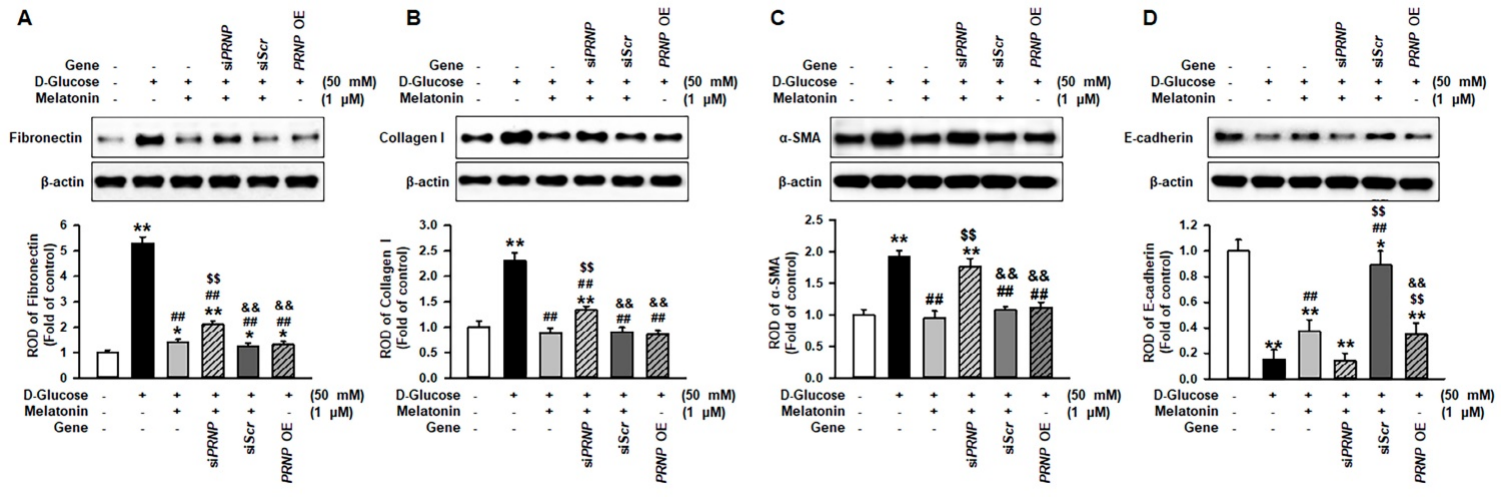
** Melatonin inhibits the change of fibrotic phenotype under high glucose conditions through the upregulation of PrP^C^.** (A-D) Expression of fibronectin (A), collagen I (B), α-SMA (C), and E-cadherin (E) in renal proximal tubule epithelial cells under high glucose conditions (50 mM) after treatment with melatonin. The levels of expression are determined relative to the expression of β-actin. Values represent the mean ± SEM (n = 3). ^*^p < 0.05, ^**^p < 0.01 vs. control, ^##^*p* < 0.01 vs. TH1 cells + D-glucose, ^$$^*p* < 0.01 vs. TH1 cells + D-glucose + Melatonin, ^&&^*p* < 0.05 vs. TH1 cells + D-glucose + Melatonin + si*PRNP*.

To determine whether melatonin regulates the expression of fibrotic markers under high glucose conditions through the upregulation of PrP^C^, pro-fibrotic markers, such as fibronectin and collagen I, and the anti-fibrotic marker, E-cadherin, were assessed in renal proximal tubule epithelial cells under high glucose conditions. Under high glucose conditions, the expression of pro-fibrotic markers was significantly increased, and the level of anti-fibrotic markers was significantly decreased. Melatonin inhibited the expression of pro-fibrotic markers and augmented the expression of anti-fibrotic markers under high glucose conditions (Figure 6A-6D). These effects were regulated by the expression of PrP^C^ (Figure 6A-6D). In addition, the overexpression of *PRNP* blocked the expression of fibrotic markers under high glucose conditions in renal proximal tubule epithelial cells that were not treated with melatonin (Figure 6A-6D). These results indicate that melatonin mitigates the expression of fibrosis-mediated markers under high glucose conditions through the upregulation of PrP^C^.

## Discussion

Hyperglycemia-induced renal fibrosis is the major cause of death from diabetic complications in patients. This study has demonstrated that melatonin protected renal proximal tubule epithelial cells against high glucose-induced fibrosis. In addition, this study also revealed the mechanism by which melatonin inhibited the expression and secretion of fibrosis-mediated ECM proteins, including fibronectin, α-SMA, and collagen I, through the PrP^C^-Smad2/7 signaling axis. Similar to the present study, recent studies also indicated that melatonin showed the protective effect on renal function against lupus nephritis through the inhibition of oxidative stress and inflammation 18, 19.

Hyperglycemia-induced inflammation and fibrosis play pivotal roles in the pathogenesis of diabetic nephropathy 20. Under high glucose conditions, the generation of ROS is increased in mitochondria because of the significant amount of glucose targets on the mitochondrial electron transport chain, leading to the generation of superoxide anions 21. Hyperglycemia-induced oxidative stress results in the increased expression of TGF-β, which leads to the deposition of ECM proteins 21. A recent review indicates that melatonin membrane receptors (MT1 and MT2) are expressed in mitochondria and melatonin plays a role in the mitochondrial targeted antioxidant through mitochondrial melatonin receptors 22. Our findings showed that melatonin decreased the production of ROS by enhancing SOD and catalase activities in renal proximal tubule epithelial cells under high glucose conditions. The level of TGF-β was also decreased by treatment with melatonin. In addition, these effects were regulated in a melatonin receptor-dependent manner. Melatonin has been reported to modulate molecular pathways of oxidative stress 8, 23, 24. Recent evidence has shown that melatonin prevents renal fibrosis in diabetic mice by activating the AMPK-PGC1α signaling pathway and recovering the function of mitochondria 25. In addition, several studies have revealed that melatonin inhibited TGF-β1 expression in lung fibrosis, liver fibrosis, and cardiac fibrosis 26-28. Furthermore, melatonin is involved in the reduction of ECM protein deposition, resulting in the improvement of fibrotic histopathology 14. These findings suggest that melatonin attenuates the expression of TGF-β1 and the deposition of ECM proteins by suppressing ROS production under high glucose conditions.

TGF-β activates the phosphorylation of receptor-associated Smads, such as Smad2 and Smad3, resulting in the stimulation of ECM protein synthesis in several cell types 29, 30. High glucose increases the phosphorylation of Smad2 and Smad3, leading to an increase in the expression of collagen I in tubular epithelial cells, mesangial cells, vascular smooth muscle cells, and vascular endothelial cells 31. In contrast to the activation of Smad2 and Smad3, the activation of TGF-β signaling also induces an increase in the expression levels of inhibitory Smads, such as Smad6 and Smad7, which suppress the phosphorylation of Smad2 and Smad3, resulting in a negative-feedback loop for TGF-β signaling 29, 30. Under high glucose conditions, the overexpression of Smad7 blocked the activation of Smad2 and Smad3 in renal and vascular cells, resulting in the inhibition of collagen I synthesis 31. Consistent with these findings, the results in this study showed that melatonin inhibited the phosphorylation of Smad2 and increased the expression of Smad7 in renal proximal tubule epithelial cells under high glucose conditions. The result was that the high glucose-induced expression of ECM proteins, including fibronectin, collagen I, and α-SMA, was decreased by treatment with melatonin. Other studies have shown that endothelial-to-mesenchymal transitions (EMT) are associated with organ fibrosis in the liver, lungs, kidneys, and intestines 32. During the development of organ fibrosis, the EMT process decreases the expression of epithelial markers, such as E-cadherin and zonula occludens-1, and increases fibroblastic markers, including vimentin, collagen I, and α-SMA 32, 33. Our data has shown that high glucose inhibited the level of E-cadherin, and melatonin improved the expression of E-cadherin under high glucose conditions. These findings indicate that melatonin inhibits the deposition of fibrosis-associated ECM proteins and increases the level of E-cadherin in renal proximal tubule epithelial cells under high glucose conditions through the regulation of TGF-β-Smad2/7 signaling pathways.

PrP^C^, which is a highly conserved and ubiquitous glycoprotein, was first recognized as a key factor of neurodegenerative disorders, such as transmissible spongiform encephalopathies or prion diseases, caused by the misfolding of PrP^C^ (PrP^Sc^) 34. However, mounting evidence has revealed that normal PrP^C^ plays important roles in physiological functions, such as stress protection, cellular differentiation, neuronal excitability, myelin maintenance, circadian rhythm, metal ion homeostasis, immunity, mitochondrial homeostasis, and interaction with several signaling pathways 15. Various studies have indicated that PrP^C^ protects cells against ROS-mediated oxidative stress through an increase in SOD activity 35-37. The overexpression of PrP^C^ has also protected against focal cerebral ischemia via the ErK1/2 signaling pathway 38. A previous study also showed that melatonin suppressed the production of ROS under oxidative stress conditions through the upregulation of PrP^C^ 35. Similar to these findings, this study has revealed that melatonin attenuated the fibrotic phenotypes by inhibiting the generation of ROS under high glucose conditions via the upregulation of PrP^C^. In addition, the overexpression of PrP^C^ in renal proximal tubule epithelial cells prevented the alteration of fibrotic phenotypes induced by high glucose. These results suggest that melatonin protects renal proximal tubule epithelial cells against high glucose-induced fibrosis through the upregulation of PrP^C^.

**Figure 7:**
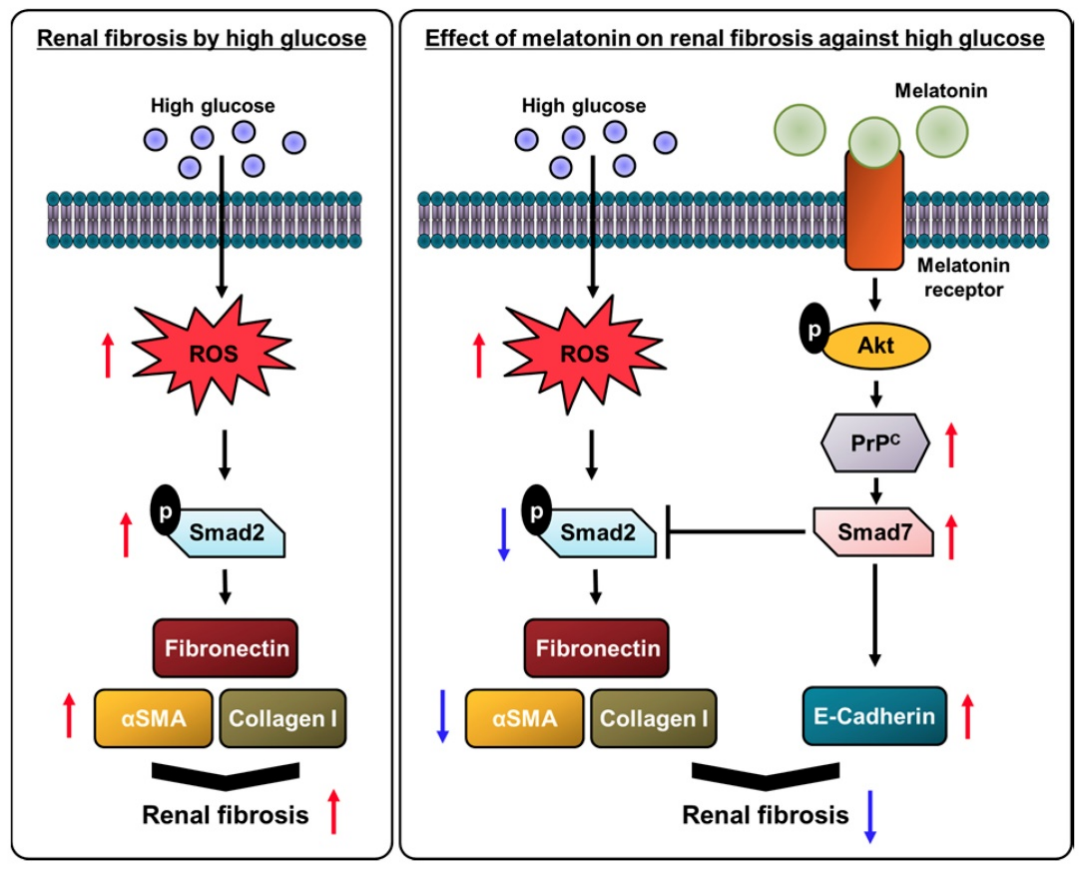
** Schematic representation of the proposed mechanism by which melatonin protects human renal proximal tubule epithelial cells against high glucose-induced fibrosis through the upregulation of PrP^C^.** Under high glucose conditions, the production of ROS is increased in human renal proximal tubule epithelial cells, resulting in cell proliferation along with a decrease in cell survival. High glucose conditions also inhibit the antioxidant activity and increase the activation of the fibrosis-mediated signaling pathway through the TGF-β-Smad2 signaling axis, which increases the level of ECM proteins, such as fibronectin, collagen I, and α-SMA. Melatonin suppresses the high glucose-induced alteration of the fibrotic phenotype through the upregulation of PrP^C^, leading to the inhibition of renal fibrosis.

Taken together, this study indicated that treatment of renal proximal tubule epithelial cells with melatonin prevented a change of fibrotic phenotype, led to the augmentation of the antioxidative effect, the reduction of TGF-β expression levels, and the decrease in deposition of ECM proteins, such as fibronectin, collagen I, and α-SMA, and increased the expression of E-cadherin, under high glucose conditions. In particular, this study has revealed the mechanism by which these protective effects of melatonin are regulated by the expression of PrP^C^ levels (Figure 7). These findings suggest that melatonin could be a therapeutic agent for diabetes patients with renal fibrosis, and the regulation of PrP^C^ levels has the potential to be a novel strategy for treating hyperglycemia-induced renal fibrosis.

## Supplementary Material

Supplementary figures and tables.
