# Supplementary material for: Melatonin Protects Human Renal Proximal Tubule Epithelial Cells Against High Glucose-Mediated Fibrosis via the Cellular Prion Protein-TGF-β-Smad Signaling Axis
Source: Int J Med Sci. 2020 May 18;17(9):1235–45. doi: 10.7150/ijms.42603 (PMC7294914; doi:10.7150/ijms.42603)
Supplement: Supplementary file 1 — Supplementary figures and tables. [file ijmsv17p1235s1.pdf]

Supplementary Figures

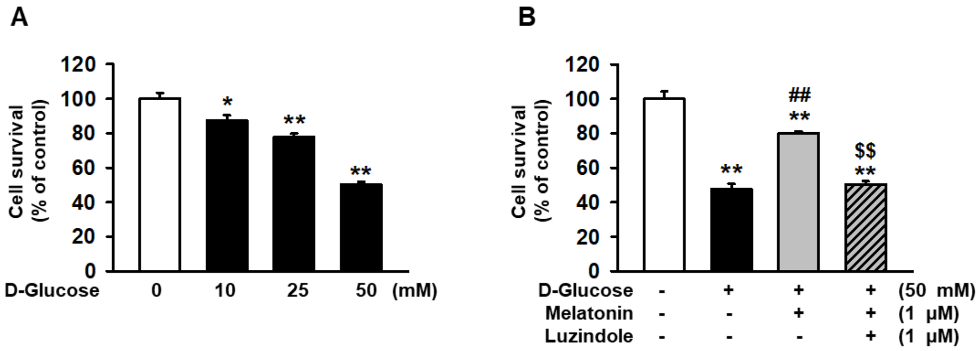

Supplementary Figure 1. Melatonin increases the survival of renal proximal tubule epithelial cells under high glucose condition.

(A) MTT assay in human renal proximal tubule epithelial cell lines (TH1) after treatment with glucose at different concentrations (0, 10, 25, and 50 mM). Values represent the mean ± SEM (n = 5). $^{*}p < 0.05$, $^{**}p < 0.01$ vs. control. (B) MTT assay in human renal proximal tubule epithelial cells after treatment with melatonin under high glucose conditions (50 mM). Luzindole acts as a selective melatonin receptor antagonist. Values represent the mean ± SEM (n = 5). $^{**}p < 0.01$ vs. control, $^{\#\#}p < 0.01$ vs. TH1 cells + D-glucose, $^{\$\$}p < 0.01$ vs. TH1 cells + D-glucose + Melatonin.

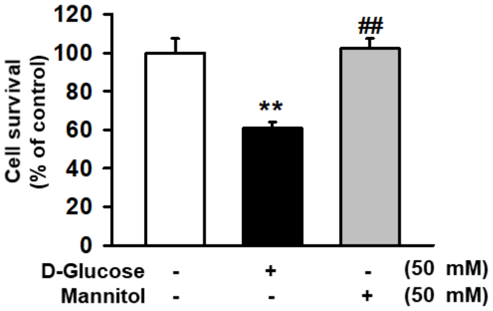

Supplementary Figure 2. Mannitol, as an osmotic control, does not affect the survival of renal proximal tubule epithelial cells.

MTT assay in human renal proximal tubule epithelial cell lines (TH1) after treatment with glucose (50 mM) or mannitol (50 mM), as an osmotic control. Values represent the mean ± SEM (n = 5). $^{**}p < 0.01$ vs. control, $^{\#\#}p < 0.01$ vs. TH1 cells + D-glucose (50 mM).
